# Persistence and irreversibility of care-demanding status: insights from long-term care claims data in Japan

**DOI:** 10.1186/s12877-025-06854-0

**Published:** 2025-12-13

**Authors:** Minamo Mikoshiba, Akira Kawamura, Toshihide Awatani, Haruko Noguchi

**Affiliations:** 1 Graduate School of Economics, Hitotsubashi University, 2-1 Naka, Kunitachi Tokyo, 186-8601 Japan; 2 Faculty of Human Sciences, Waseda University, Tokorozawa, Saitama Japan; 3 Department of Family Medicine, Kochi Medical School, Kochi University, Nankoku, Kochi Japan; 4 Faculty of Political Science and Economics, Waseda University, Shinjuku, Japan; 5 Waseda Institute of Social and Human Capital Studies (WISH), Waseda University, Shinjuku, Japan

**Keywords:** Disability, Mortality, Cognitive impairment, First-order Markov chain, Monte Carlo simulations

## Abstract

**Background:**

Profiling disability and mortality risks in older adulthood is essential for government planning, resource allocation, and care services provision. This study showed trajectory of disability and mortality risks using objective measures derived from administrative long-term care insurance (LTCI) claims data in Japan.

**Methods:**

This cohort study used longitudinal data from LTCI claims, linked with death certificates, and supplemented with population data for non-LTCI eligible individuals, from 2006 to 2018. The dataset comprised 30,347,066 older adults’ records aged 65–94 eligible for LTCI within 1912–1951 birth cohorts (7,221,142 unique individuals). The definition of disability was based on LTCI claims data, quantified by standard hours of care. One-year interval transition probabilities of disability and mortality, categorized by age, sex, and care demanded level, were estimated using a first-order Markov chain.

**Results:**

The participants had a mean age of 83.8 years (SD = 6.8), with 69.5% being female. Eligibility rates sharply increased in the mid-70s, reaching at 66.8% (81.1%) at 94 years of age for males (females). Probabilities of transitioning to no-disability were almost zero, while maintaining the same level of care showed notably high probabilities, ranging from 60.3% to 99.2% across all ages, sexes, and current care levels. Transition from light-cognitive to heavy-cognitive status ranged between 19.1% and 24.5%. Monte Carlo simulations demonstrated almost perfect predictability of disability and mortality risks in older adults, with maximum difference, 3.2 (2.6) ppt for disability (mortality).

**Conclusions:**

Care service demand proves mostly irreversible. Measures should focus on preventing worsening disability levels in older adults without disabilities or with only light disabilities, with particular emphasis on preventing cognitive impairment.

**Supplementary Information:**

The online version contains supplementary material available at 10.1186/s12877-025-06854-0.

## Introduction

Global aging is one of the most urgent and critical challenges of the 21 st century. According to the World Population Prospects, the population aged 65 and above is expected to double by 2050, reflecting a significant increase in life expectancy [1]. Although people are living longer, not everyone remains healthy in older adulthood without their daily activities significantly limited. The United Nations reports that more than 46% of those aged 60 and above have disabilities [2]. Additionally, the World Health Statistics 2022 shows a growing number of years lived with a disability in older adulthood [3]. 

Age is strongly associated with increased risks of physical and cognitive disabilities, long-term care status (LTC-status), and mortality [4–6]. Profiling the trajectory patterns of disability and mortality rates across different ages provides valuable insights for older adults, their families, researchers, and practitioners. Such insights can help older adults and their informal caregivers in making care plans for later life, influence decisions made at younger ages regarding savings and private long-term care insurance enrolment, and help governments budget for long-term care services.

Numerous studies have already tackled this issue in high- and middle-income countries, finding that the prevalence of chronic disability in older adulthood has declined, due to refined nutrition, sanitation, and education [7–12]. However, many follow-up studies have reported that the probabilities of disability and disease increase with age [10, 13, 14]. Most of these studies rely on nationally representative survey data [15–17] or administrative claims data, such as Medicare in the United States [18, 19]. Some studies examine short-term disability trajectories at the end of life, unveiling distinct patterns across clinical conditions leading to death [6, 20–22]. Nonetheless, to the best of our knowledge, no study has demonstrated the disability and mortality trajectory using almost nationally representative long-term administrative claims data. This gap exists because only a few countries have public and mandatory long-term care insurance (LTCI) systems, leading to limited data accessibility.

This study uses data from Japan’s LTCI system, which covers the entire population aged 40 and above, to estimate one-year interval transition risks of disability and mortality by age, sex, and LTC-status among older adults aged 65–94. While survey data may include information that impacts disability risks [23, 24], they can be subject to self-selection and response biases [25] and often have limited sample sizes [9]. In contrast, administrative data, although containing fewer variables, provide a large sample size and avoids these biases. Japan’s super-aged society offers a blueprint for long-term care policies in other developed countries with similar insurance systems.

Most studies define older adults as disabled when they need help performing activities of daily living (ADLs) and instrumental activities of daily living (IADLs) or demonstrate physical and cognitive limitations or functional loss, often accompanied by comorbidities such as cardiovascular diseases or diabetes [9, 11]. However, there is no globally accepted standard for measuring disability, likely because previous studies have applied various statistical approaches to self-reported survey data [11]. This study employs a unique, objective and scientifically based measure introduced by Japan’s LTCI system in 2000. It assesses individual care levels and actual use of care services as determined by trained professionals, rather than relying on self-reported disability status [26]. 

## Japan's long-term care insurance

With the implementation of LTCI in 2000, Japan became one of the first countries to develop mandatory public insurance schemes for long-term care both within facilities and at home. The official purpose of introducing Japan’s LTCI was to assist older adults who need care “to maintain dignity and an independent daily life routine according to each person’s own level of abilities.” [27]. The LTCI introduced clear eligibility standards for long-term care use, based on the tree-regression model [28] for estimating the time required to support ADLs, IADLs, behavioral and psychological symptoms of dementia (BPSD), functional training, and medical care. This model utilized data from a time study conducted in 1995 [29, 30] and was refined through pilot studies and feedback from healthcare and long-term care professionals up to 2000. Table 1 displays the estimated minutes required for each care activity according to the tree-regression model based on the time survey. To calculate the standard hours of total care required, a trained local government official evaluates a 74-item questionnaire on ADLs, IADLs, BPSD, functional training, and medical care use. This evaluation categorizes each older adult into one of eight care levels using a computer algorithm and an expert committee. These care levels include independent, support-required level (SL) 1–2, and care-required level (CL) 1–5. If classified as independent, older adults are ineligible for LTCI. SL refers to recipients who live independently but require assistance with IADLs. CL1-2 care recipients may be able to live alone with partial assistance for basic activities but exhibit lower levels of thinking and comprehension and may have some behavioral challenges. CL3-5 care recipients require full support for daily activities. Table 2 presents the standard hours for each care level.

Eligibility for LTCI is determined purely based on the level of care required, regardless of socioeconomic attributes, without means tests [31]. The LTCI claims data include recipients of welfare benefits who are eligible for free long-term care assistance, comprising approximately 6.3% of all recipients in 2020. Therefore, the LTCI system provides comprehensive information on care levels for the entire population of older adults in need of care, making it an ideal environment for examining the transition probabilities of care levels without sample selection bias.

The insured population includes individuals aged 65 and over, as well as those aged 40 to 64 who are covered by the health insurance system. Premiums are also paid by those aged 40 to 64, as the risk of disability increases after the age of 40 due to age-related diseases, and their parents are at a higher risk of needing care [32]. Those aged 65 and over are eligible for LTCI services only if they receive certification of needing care, regardless of the underlying cause. In contrast, individuals aged 40 to 64 are eligible for LTCI services only if they are certified due to specific age-related diseases. This study focuses on individuals aged 65 and over.

## Methods

### Data sources

We used the “Statistics of Long-term Care Benefit Expenditures (SLBE)” and “Vital Statistics-Death Certificates (VS-DC),” conducted by the MHLW from 2006 to 2018. The SLBE is a nationwide administrative data comprising LTCI claims of all residents in Japan eligible for LTCI. It includes information such as sex, age, municipality of residence, insurance identification number, level of care demanded, dates of qualification and disqualification, and service utilization. The VS-DC provides information on date of birth, municipality, and date of death.

Additionally, we complemented these individual-based data with two aggregated datasets: “the Japanese Mortality Database (JMD),” provided by the National Institute of Population and Social Security Research (NIPSSR), and “Population, Vital events and Households Derived from Basic Resident Registration (BRR),” provided by the Ministry of Internal Affairs and Communications (MIC).

### Study population

**Fig. 1:**
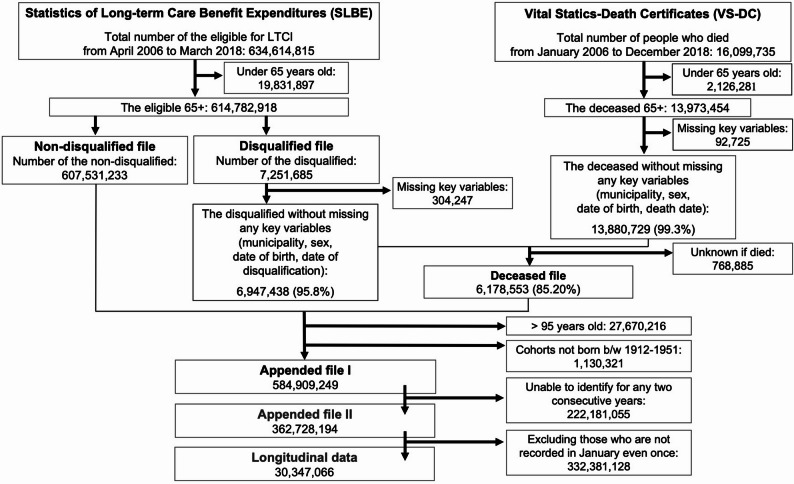
Flowchart of how to construct the longitudinal data

The inclusion criteria used in this study are as follows: (i) individuals aged 65–94 years, (ii) those for whom the date of death among disqualified people can be identified, (iii) those who can be traced for any two consecutive years from April 2006 to March 2018, and (iv) those who were recorded in January during the observational period. Figure 1 illustrates the flowchart depicting the process of constructing longitudinal data for each eligible individual under LTCI during the observation periods.

Initially, we excluded individuals below 65 years of age, totaling 19,831,897 records (out of 634,614,815 records) from the SLBE and 2,126,281 records/individuals (out of 16,099,735 records/individuals) from the VS-DC. Subsequently, the SLBE was divided into two files: the “Disqualified file” (7,251,685 records) and the “Non-disqualified file” (607,531,233 records). The SLBE does not include the death date; instead, it only records the date of disqualification due to death, relocation to a different municipality, achieving ineligibility, or independence. Notably, relocation to a different municipality is recorded as disqualification because an identification number is assigned when individuals move. We conducted a deterministic linkage between the “Disqualified file” and VS-DC using identifiers such as municipality of residence, sex, birth date, disqualification date, and death date to determine the timing of death (resulting in the “Deceased file,” 6,178,553 records). This linkage process is necessary because municipal offices in Japan disqualify the deceased from LTCI upon receiving notice of death and the death certificate, thereby recording the date of disqualification.

In the next step, we appended the “Non-disqualified” and “Deceased” files (613,709,786 records). We then excluded individuals over 95 years of age and those not born between 1912 and 1951 (excluded 28,800,537 records, 4.7% of the records), as the sample size by age was insufficient to estimate stable transition probabilities. This resulted in “Appended file I” (584,909,249 records). We also excluded individuals who could not be traced for two consecutive years, resulting in the “Appended file II,” 362,728,194 records). We then constructed longitudinal data spanning from January 2007 to January 2018, extracting data from January of each year. This process yielded one-year interval longitudinal data (30,347,066 records) for the 1912 to 1951 cohort (7,221,142 unique individuals).

Unfortunately, the SLBE does not cover individuals who are ineligible for LTCI. Accordingly, when computing the transition probabilities from independence to any care level, we used the estimated number of non-disabled individuals by age and sex for the same cohort as the above longitudinal data, based on the BRR and JMD. Supplementary Methods 1–2 and Supplementary Fig. 1 detail the calculation of the transitions from independence to other care level.

### Estimation methods

First, we classified LTC-status into four categories based on its eight levels, as described in the previous section: no-disability if those ineligible for LTCI, light if the levels range from SL1 to CL2, heavy if the levels range from CL3 to CL5, and death if deceased. The longitudinal data comprises no-disability (0.02%), light (53.2%), heavy (36.5%), and death (10.2%).

Second, to examine the persistence of LTC-status, we estimated a 4 × 4 transition matrix for LTC-status for each sex from age 65 to 94 by computing a first-order Markov chain, resulting in 12 transition patterns. The total population for which municipalities provide long-term care claims data to the SLBE represents 79.3% of the total population aged 65 and over in Japan. To correct for population variation in the cohort and municipalities, we calculated weighted averages of transition probabilities by age and sex using each year’s municipal population data from the BRR and JMD, spanning from 2007 to 2018. Supplementary Methods 3 and Supplementary Fig. 2 detail the missing municipalities in the SLBE.

Third, to validate the accuracy of the computed transition probabilities in representing the distribution of each LTC-status based on the raw data, we conducted Monte Carlo simulations using the transition probabilities. We conducted 500,000 simulation trials to calculate the rates of individuals in each LTC-status and the mortality risks from age 65 to 94. Detailed explanations of the Monte Carlo simulations are presented in Supplementary Methods 4.

### Additional analysis

We narrowed our focus to individuals utilizing care services covered by LTCI, further classifying them based on their service usage. Recipients were classified as cognitive service users if they used any dementia-related service, or as physical service users otherwise. This classification resulted in five LTC-status categories: light-physical, light-cognitive, heavy-physical, heavy-cognitive, and death. This classification generated a 5 × 5 transition matrix for LTC-status (20 transition patterns). Older adults with no-disability were automatically excluded from this analysis due to LTCI ineligibility. Therefore, the total and unique observations for this analysis were 21,108,348 and 648,328, respectively, both fewer than in the previous dataset. These categories comprised light-physical (51.4%), light-cognitive (3.2%), heavy-physical (34.7%), heavy-cognitive (5.0%), and deceased individuals (5.7%). Subsequently, we calculated transition probabilities for each sex from age 65 to 94 using a first-order Markov chain. All analyses were performed using R Statical Software, version 4.2.1 (R Core Team 2022).

## Results

### Disability risk in older adults

**Fig. 2:**
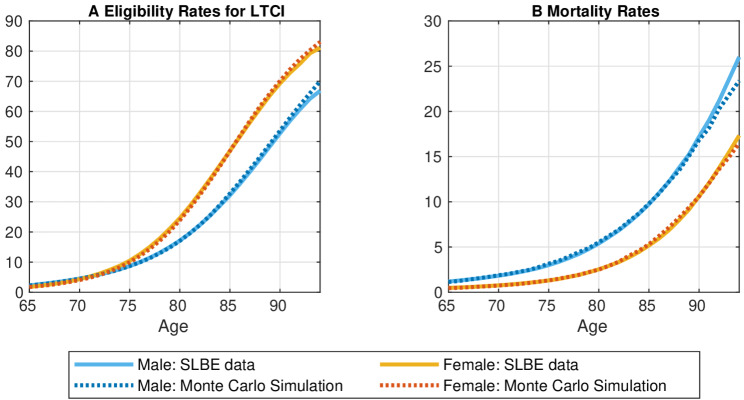
Disability and mortality risks by age and sex (raw and simulation). Note: We compute both the average eligibility rates and the average mortality rates from the same raw data on cohort, age, and sex that we use when computing the transition probabilities. Since the SLBE does not cover individuals who are ineligible for long-term care insurance, we estimate the non-disabled older adult population by cohort, sex, and age, using only raw data with positive estimated populations when calculating transition probabilities. The simulations are conducted by the Monte Carlo method

Figure 2A illustrates the average rates of older adults eligible for LTCI by age and sex, alongside the expected rates determined using Monte Carlo simulations. On average, the eligibility rates remain below 10% until the mid-70s for both sexes. However, the rates grow shapely after the mid-70s, reaching 66.8% for males and 81.1% for females by age of 94. Until the mid-70s, the rates are slightly higher for males than females, but this trend reverses after the mid-70s with the rates for females significantly exceeding that for males. As depicted in Fig. 2A, the simulated rates effectively capture the real average rates.

Although the average profiles provide insights into the expected risk of becoming disabled, the degrees of risk may be heterogeneous among older adults. Typically, the severity of LTC-status increases with age. These findings indicate that the rates worsen more rapidly among females, as depicted in Supplementary Fig. 3.

### Persistence of disability risk

**Fig. 3:**
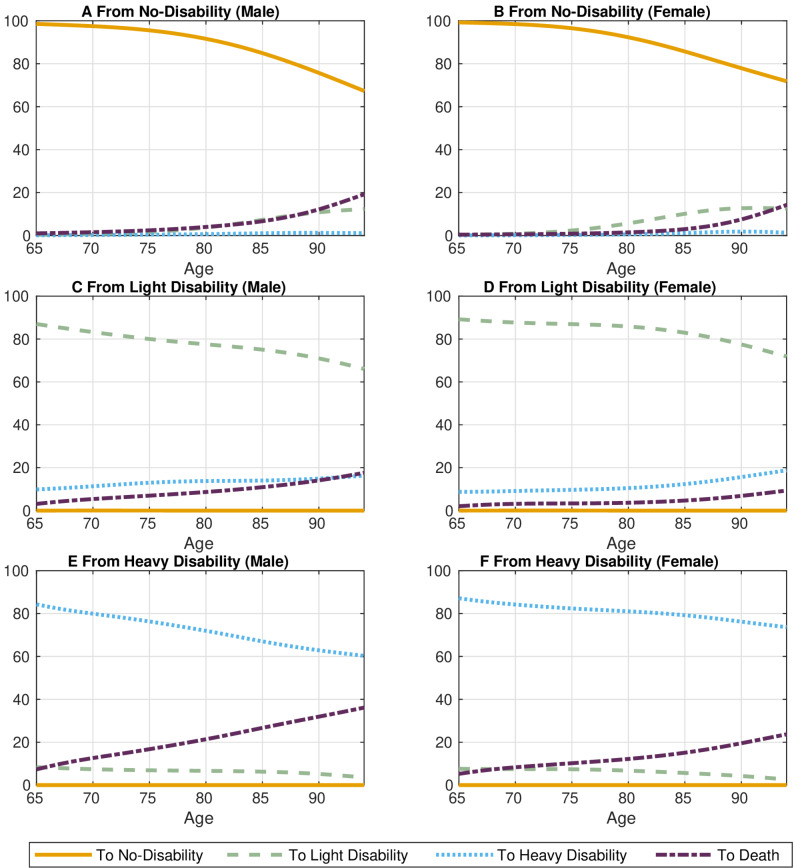
Transition probabilities by current LTC-status, age and sex

We examined the persistence of LTC-status by computing the transition matrix of LTC-status by age and sex based on a first-order Markov chain. Figure 3 illustrates the transition probabilities across all ages by sex and current LTC-status. Our analysis reveals a high persistence of LTC-status, indicating that the LTC-status is most likely to remain unchanged in the subsequent period across all ages, sexes, and current LTC-status. For example, at age 75, the probabilities of staying in the same LTC-status range from 76.3% to 95.6% for males and 82.4% to 96.5% for females. Moreover, once an older adult becomes disabled and eligible for LTCI, the probability of transitioning to a no-disability status is almost zero, regardless of age or sex.

The future risk of disability increases with the severity of current LTC-status and age. The more severe the current LTC-status, the higher the probability of transitioning to a more severe status in subsequent periods. Additionally, the risk of moving to a more severe LTC-status escalates with age.

### Mortality risk

Figure 2B presents the average probabilities of older adults not surviving the following year by age and sex, alongside the expected probabilities derived from the Monte Carlo simulation. We confirmed that the simulated probabilities effectively capture the observed data. Overall, the mortality risk is substantially higher for older males than for older females regardless of age, with sex-based disparities widening as age increases.

Figure 3, however, illustrates the death probabilities vary not only by age and sex but also by current LTC-status. The risk of not surviving the following year increases with age, given the current LTC-status. Generally, the more severe the LTC-status, the higher the mortality risk across ages. Nevertheless, once individuals surpass the age of 90, the mortality risk for those with no-disability becomes higher than for those with light-disability.

### Risk of cognitive and physical disabilities

**Fig. 4:**
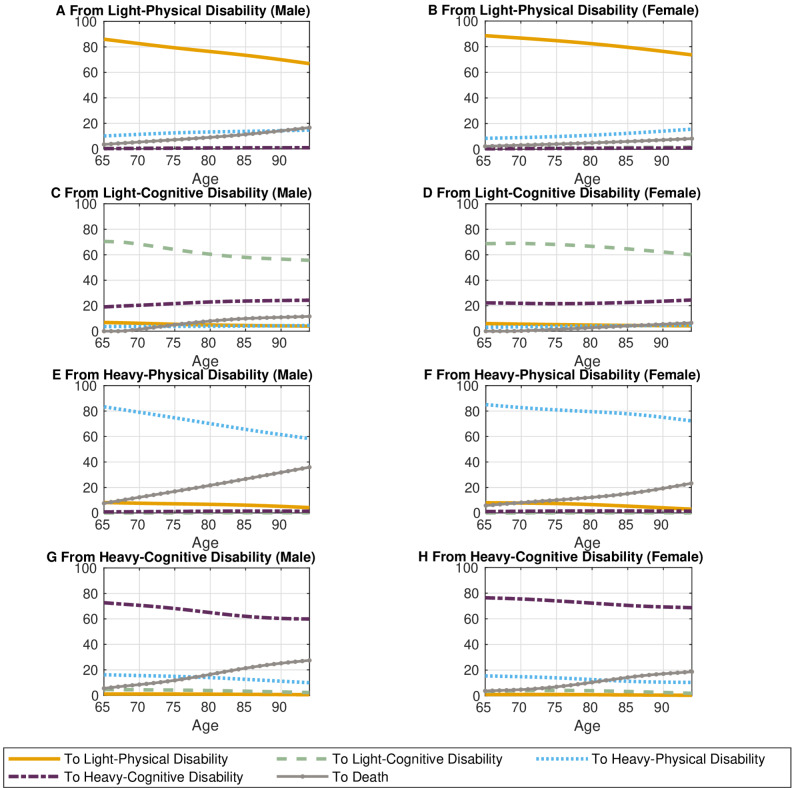
Transition probabilities by current cognitive and physical LTC-status, age, and sex

Figure 4 illustrates the estimated transition probabilities for the following year, conditional on the current LTC-status by age and sex, using a first-order Markov chain, when we further classified disability status into two groups: cognitive and physical service users. Consistent with the results shown in Fig. 3, we generally observed high persistence of LTC-status even after distinguishing the type of services, regardless of age or sex.

As shown in Fig. 4, cognitive status is less persistent than physical status, with 20–25% of individuals with light-cognitive status progressing to heavy-cognitive status (Fig. 4C and D) compared to 10–15% of those with light-physical status developing heavy-physical status (Fig. 4A and B). Meanwhile, regardless of whether the status is cognitive or physical, once an older adult develops a heavy status, their risk of disability rarely improves (Fig. 4E, F, G and H). Additionally, the probability of transitioning from heavy-cognitive to heavy-physical status is higher than the probability of death until around age 80 for males and 85 for females; this trend subsequently reverses (Fig. 4G and H).

Figure 4 also shows the mortality rates by current physical and cognitive LTC-status, age, and sex computed using a first-order Markov chain. We confirmed that mortality risks are significantly higher for males than females at any age, and that the heavier the LTC-status, the higher the probability of death across all ages, consistent with Fig. 3. Notably, Fig. 4 reveals that, irrespective of the severity of LTC-status, older adults with physical disabilities are more likely to die than those with cognitive LTC-status across all ages and sexes. These findings imply that physical disabilities more seriously impact mortality than cognitive disabilities, given the level of LTC-status.

## Discussion

This study examined the trajectories of disability and mortality risk among individuals aged 65 and 94, utilizing objective measures of long-term care needs derived from administrative LTCI claims data in Japan.

Regarding disability risk, our findings indicate that the demand for care increases with age, rising sharply after the age of 75, and that the risk of disability is highly persistent and irreversible. Moreover, older adults with cognitive disabilities are more likely to transition to a severe status that older adults with physical disabilities. In terms of mortality, the mortality rate is higher for older individuals and those with a greater demand for care. The Monte Carlo simulations demonstrate that the trajectory patterns of disability and mortality rates in older adulthood can be predicted with high accuracy using population-based administrative claims data, which covers approximately 80% of the population aged 65 years and older in Japan.

The persistence and irreversibility of LTC-status estimated in this study align with trajectories observed in previous studies [6, 15–22]. The novelty of our study lies in its use of data that are (almost) nationally representative [33, 34, 36] and in analyzing the level of care certified by the LTCI system as a proxy for functional ability [6, 15–19, 21, 22]. Additionally, several studies have corroborated the robustness of our findings. The high persistence of LTC-status is also confirmed in transitions among the seven levels of care needs defined by LTCI [35], as detailed in Supplementary Methods 5 and Supplementary Table 1. In studies utilizing administrative data from specific municipalities [36, 37], the transition rate for individuals eligible for LTCI who are later certified as independent is close to zero, consistent with the results of our study.

Although the primary objective of the study is to profile disability among older adults, discussing potential factors underlying our findings is worthwhile. First, the rapid increase in disability and mortality risks around age 75 may reflect a sharp rise in morbidity among individuals aged 75 and older. It is well-known that the risks of disability and mortality are often associated with the onset of diseases such as cardiovascular diseases, chronic obstructive pulmonary disease (COPD), and tuberculosis [38]. According to the Patient Survey (another nationally representative survey) [39], incidence rates for nervous system disorders and bone fractures are approximately two to three times higher in the 75–79 age group compared to the 65–69 age group.

Second, the sex-specific prevalence rates of these diseases may partially explain in the heterogeneity in disability and mortality risks across sexes [40]. Disability risk is nearly equivalent or slightly higher for males until the mid-70s. However, it escalates more rapidly for females with age. For example, until the mid-70s, incidence rates for ischemic heart disease, cerebrovascular disease, and nervous system disorders are more than twice as high in males as in females, but this trend reverses beyond that age. Whereas the mortality rates are consistently higher for males than for females across all ages and LTC-status, the incidence rates of diseases such as malignant neoplasms, diabetes, COPD, tuberculosis, and pneumonia remain higher in males than in females across all age groups.

Third, it is notable that individuals aged 90 and older with no-disability status face a higher risk of death compared to those with light disability status. This phenomenon may be attribute to the distinct structure of Japan’s LTCI and health insurance systems. Under the Japanese health insurance system, hospitalized patients are ineligible to receive long-term care services under LTCI. Additionally, it is worth noting that approximately 72% of Japanese individuals pass away in hospitals [41]. 

Our study may still serve as a reference point for developed countries with similar universal long-term care systems, though our findings may not be universally generalizable across different societal context. Given the high persistence and irreversibility of LTC-status, older adults with no-disability or light-physical status should take preventative measures, such as maintaining appropriate nutrition intake and engaging in moderate exercise, to reduce the risk of progression in their LTC-status and improve stability over time. Preventing cognitive impairment is especially crucial for those with cognitive status, as they face a heightened risk of worsening their disability.

For a long-term perspective on economic and fiscal sustainability, quantifying the overall demand for long-term care, as well as the severity and duration of disability risk among older adults, is essential. By measuring disability and mortality risks, this study provides a foundation for quantitatively assessing the extent of the burden on households and the government. The steep increase in disability risk after age 75, as demonstrated in this study, suggests a corresponding rise in the demand for care as the population of older adults grows. Given the high persistence and irreversibility of LTC-status, individuals who require care and their families may face a prolonged burden until the end of life. Quantifying and sharing information on disability risk allows older adults and their families to make informed long-term care plans and consider proactive measures such as precautionary savings and private insurance options.

Our study has some limitations. First, we examine annual changes observed each January throughout the study period, based on the requirement to update care levels at six month or one-year intervals. However, this approach may overlook individuals who transition to a no-disability status before January, as the SLBE data do not track status changes following a return to no-disability. Consequently, we cannot distinguish between those who remain in a no-disability state and those who may have passed away. As such, caution is warranted in interpreting our results. Further research should aim to link medical care and long-term care claims data with mortality records to enable more detail and comprehensive analyses of transitions from morbidity to disability and eventually to mortality in older adults.

Second, previous research using data from a specific municipality has highlighted potential manipulation of standard care hours, possibly to prevent recipients from being assigned to a lower care level than previously certified [42]. Although the SLBE data in our study are nationwide administrative claims, if such manipulation occurs at national level, it may lead to an overestimation of LTC status irreversibility. This remains an area for future research, as our dataset does not include standard care hours at the time of care level certification.

**Table 1:** Estimated minutes and standard hours of care under the LTCI system in Japan estimated minutes for care activity based on assessment strategy

(a) Estimated minutes for care activity based on assessment strategy			
Classification		Care activity	Estimated minutes for each care activity (min)
ADLs	Eating	Food intake, swallowing, etc.	1.1–71.4
	Excretion	Cleaning up after elimination, putting on/taking off pants, etc.	0.2–28.0
	Mobility	Mobility in daily life.	0.4–21.4
	Cleanliness	Dressing and undressing, bathing, washing, etc.	1.2–24.3
IADLs		Household chores, such as laundry and cleaning.	0.4–11.3
BPSD		Searching for wandering individuals, cleaning up after filthy behavior, etc.	5.8–21.2
Functional training		Walking training, training for daily living, etc.	0.5–15.4
Medical care		Administration of infusions, treatment of bedsores, etc.	1.0–37.2.0.2

The source of table is from website of the Ministry of Health, Labour andWelfare. Website: https://www.mhlw.go.jp/topics/kaigo/nintei/dl/text2009411.pdf (in Japanese) (Accessed July 15, 2024)

**Table 2:** Estimated minutes and standard hours of care under the LTCI system in Japan estimated minutes for care activity based on assessment strategy

(b)Standard hours of eight levels of care		
Care level	Approximate physical condition	Estimated minutes for total care demanded (min)
Ineligible or independent	Able to perform basic ADLs, such as walking and getting up on their own, and capable to perform some IADLs, such as taking medications and using the telephone.	Less than 25
SL1	Able to eat and defecate on their own for the most part, but require assistance in some aspects of ADLs, such as standing up, and need support to alleviate or prevent worsening of the condition.	25-Less than 32
SL2	Able to perform ADLs have declined slightly from SL1 and require some support or partial long-term care.	32-Less than 50
CL1	Able to perform ADLs have partially decreased from SL2 and require some assistance to lead a daily life.	32-Less than 50
CL2	Require some assistance with eating and toileting, and support when standing up and walking. Cognitive ability and memory may deteriorate.	50-Less than 70
CL3	Demand partial assistance with eating and toileting. Cannot stand up by themselves. Need full assistance with bathing, dressing, and undressing. Some problematic behaviors and cognitive and comprehension deficits may be observed.	70-Less than 90
CL4	Occasionally need assistance with eating, and require full assistance with toileting, bathing, and dressing. Difficult to lead daily life without care. Many behavioral problems and a general loss of comprehension.	90-Less than 110
CL5	Almost impossible to lead daily life without care, such as being unable to eat or defecate on one’s own. Exhibit many behavioral problems and a decline in comprehension.	110 and more

The source of table is from website of the Ministry of Health, Labour and Welfare. Website: https://www.mhlw.go.jp/stf/seisakunitsuite/bunya/hukushikaigo/kaigokoureisha/nintei/index.html (in Japanese) (Accessed July 15, 2024)

## Supplementary Information

Supplementary Material 1

## Data Availability

The datasets used and analyzed during the current study are available from the SLBE and VS-DC, conducted by the MHLW (Ministry of Health, Labour and Welfare) from 2006 to 2018. But restrictions apply to the availability of these data, which were used under official approval by the MHLW for the current study, and so are not publicly available. Data are however available with permission of the MHLW.

## Abbreviations

LTC: Long-Term Care
LTCI: Long-Term Care Insurance
ADLs: Activities of daily living
IADLs: Instrumental activities of daily living
BPSD: Behavioral and psychological symptoms of dementia
MHLW: Ministry of Health, Labour and Welfare
SL: Support-required level
CL: Care-required level
SLBE: Statistics of Long-term Care Benefit Expenditures
VS-DC: Vital Statistics-Death Certificates
JMD: The Japanese Mortality Database
NIPSSR: National Institute of Population and Social Security Research
BRR: Basic Resident Registration
MIC: Ministry of Internal Affairs and Communications
COPD: Chronic obstructive pulmonary disease
