# Supplementary material for: Persistence and irreversibility of care-demanding status: insights from long-term care claims data in Japan
Source: BMC Geriatr. 2025 Dec 13;26:139. doi: 10.1186/s12877-025-06854-0 (PMC12860092; doi:10.1186/s12877-025-06854-0)
Supplement: Supplementary file 1 — Supplementary Material 1 [file 12877_2025_6854_MOESM1_ESM.docx]

**Supplementary Material: Persistence and Irreversibility of Care-demanding Status: Insights from Long-Term Care Claims Data in Japan**

**Supplementary Methods 1. A First-order Markov Chain**

To demonstrate the profile of disability in older adulthood, we computed transition probabilities for LTC-status by sex from age 65 to 94 by using a first-order Markov chain (Chung 1967; Robinson 1996).

We defined the state space $X$ as $\left\{ x_{1},x_{2},x_{3},x_{4} \right\}$, where$x_{1},x_{2},x_{3}$ and $x_{4}$ correspond to no-disability, light, heavy, and death, respectively. For each sex $s \in\left\{ m,f \right\}$ and age $j \in\left\{ 65,\ldots,94 \right\}$, we computed a transition $4 \times4$ matrix $P_{s,j}$. The element $P_{s,j}\left( a,b \right)$ represents the probabilities of transitioning from the current state $a \in X$ to the next state $b \in X$ in one year for each sex and age.

$P_{s,j}\left( a,b \right)=\text{Pr}\left( x_{s,j+1}=b | x_{s,j}=a \right)$

The matrix $P_{s,j}$ satisfies the following assumptions:

1. For each sex $s$ and age $j$, the matrix $P_{s,j}$ satisfies

$\sum_{n=1}^{4} P_{s,j}\left( a,x_{n} \right)=1$ for all $a\in X$

2. For each sex $s$ and age $j$, death $x_{4}$ is an absorbing state.

$P_{s,j}\left( x_{4},x_{1} \right)=P_{s,j}\left( x_{4},x_{2} \right)=P_{s,j}\left( x_{4},x_{3} \right)=0,$ $P_{s,j}\left( x_{4},x_{4} \right)=1$

A first-order Markov chain simplifies the process of LTC-status transitions, assuming that the probability of moving to the next state in one year depends solely on the current state, independent of previous sequences leading to the current state. Using this chain, we also computed the probabilities of moving from one state to another over two years.

$$P_{s,j}^{\left( 2 \right)}\left( a,b \right)=\text{Pr}\left( x_{s,j+2}=b | x_{s,j}=a \right)$$

$= \sum_{n=1}^{4} \text{Pr}\left( x_{s,j+2}=b | x_{s,j+1}=x_{n} \right)\text{Pr}\left( x_{s,j+1}=x_{n} | x_{s,j}=a \right)$

$=\sum_{n=1}^{4} \text{Pr}\left( x_{n},b \right)\text{Pr}\left( a,x_{n} \right)$

By iterating this process, we computed the probabilities of transitioning between states over $k\geq1$ years.

$$P_{s,j}^{\left( k \right)}\left( a,b \right)=\text{Pr}\left( x_{s,j+k}=b | x_{s,j}=a \right)$$

The first-order Markov chain provides a straightforward estimation of LTC-status transitions. Higher-order Markov chain ($k>1$) would complicate the computation of these transitions.

**Supplementary Methods 2. Calculation of the transition probabilities of LTC-status**

The SLBE excludes individuals who are ineligible for LTCI due to being independent without any ADLs or IADLs. To calculate transition probabilities from independence to any LTC-status level, we estimated the national-scale population $N$ across 12 transition patterns for LTC-status over two periods using the BRR and JMD. In essence, we estimated the national-scale transition population $N_{s,i,j}\left( a,b \right)$ from the current state $a \in\left\{ x_{1},x_{2},x_{3} \right\}$ at age $j$ to the next state $b\in X$ at age $j+1$ for each cohort $i$ and sex $s$. Supplementary Figure 1 shows a flowchart illustrating the methodology used to estimate these national-scale transition populations and the corresponding transition probabilities for moving between different states over two periods.

Firstly, we estimated the national-scale transition population from both light and heavy LTC-status to any level of LTC-status, including transitions from light to no-disability, light, heavy, and death, and from heavy to no-disability, light, heavy, and death. The population $N_{s,i,j}\left( a,b \right)$ transitioning from state $a\in\left\{ x_{2},x_{3} \right\}$ at age $j$ to state $b\in X$ at age $j+1$ for each cohort $i$ and sex $s$ is estimated as:

$N_{s,i,j}\left( a,b \right)=N_{s,i,j}\left( a \right)P_{s,i,j}\left( a,b \right)$ for $a\in\left\{ x_{2},x_{3} \right\}$ and $b\in X$

where $N_{s,i,j}\left( a \right)$ represents the estimated national-scale population in the state $a$ at age $j$ for cohort $i$ and sex $s$ using the BRR and “Appended file I.” We complied “Appended file I” for each January from 2007 to 2018 into a national-scale database for light and heavy statuses across cohorts, sexes, and ages, using the BRR to adjust for missing municipalities in the SLBE and population variations across cohorts and municipalities. $P_{s,i,j}\left( a,b \right)$ denotes the transition probabilities from the state $a$ at age $j$ to state $b$ at age $j+1$ for each cohort $i$ and sex $s$ using one-year interval longitudinal data. We calculated the weighted averages of these transition probabilities from light and heavy statuses to any LTC-status by cohort, sex, and age using the BRR from 2007 to 2018 to correct for missing municipalities in the SLBE.

Secondly, we computed the national-scale population transition from independence to any LTC-status level, such as transitions from no-disability to no-disability, light, heavy, and death, using death rates $d_{s,i,j}$ and population size $T_{s,i,j}$ by cohort $i$, sex $s$, and age $j$ in the JMD between 2007 to 2018. The national-scale population transition from independence to any LTC-status is estimated as follows.

${N_{s,i,j}\left( x_{1},x_{4} \right)=d}_{s,i,j}T_{s,i,j}-(\sum_{n=2}^{3} N_{s,i,j}(x_{n},x_{4}))$

$N_{s,i,j}\left( x_{1},x_{1} \right)=(T_{s,i,j}-\sum_{n=1}^{3} N_{s,i,j}(x_{n},x_{4}))(1-\sum_{n=1}^{4} (N_{s,i,j}\left( x_{2},x_{n} \right)+N_{s,i,j}(x_{3},x_{n}))/T_{s,i,j})-(\sum_{n=2}^{3} N_{s,i,j}(x_{n},x_{1}))$

${N_{s,i,j}\left( x_{1},x_{2} \right)=(T}_{s,i,j}-\sum_{n=1}^{3} N_{s,i,j}\left( x_{n},x_{4} \right))(\sum_{n=1}^{4} N_{s,i,j}\left( x_{2},x_{n} \right)/T_{s,i,j})-(\sum_{n=2}^{3} N_{s,i,j}(x_{n},x_{2}))$

${N_{s,i,j}\left( x_{1},x_{3} \right)=(T}_{s,i,j}-\sum_{n=1}^{3} N_{s,i,j}\left( x_{n},x_{4} \right))(\sum_{n=1}^{4} N_{s,i,j}\left( x_{3},x_{n} \right)/T_{s,i,j})-(\sum_{n=2}^{3} N_{s,i,j}(x_{n},x_{3}))$

Thirdly, we excluded cohorts, sexes, and ages with negative population, and calculated weighted averages of transition probabilities $P_{s,j}$ by sex $s$ and age $j$ using the JMD from 2007 to 2018 to adjust for population variations in the cohort.

**Supplementary Methods 3. Missing Municipalities in the SLBE**

The SLBE is a nationwide administrative data that comprises long-term care claims from all residents of Japan who are eligible for LTCI. However, some municipalities do not submit long-term care claims data to the SLBE. The population for which municipalities provide long-term care claims data to the SLBE accounts for 79.3% of the total population aged 65 and over in Japan. Supplementary Figure 2 illustrates the rates of the population for which municipalities provide long-term care claims data to the SLBE relative to the total population aged 65 and over in each prefecture. In most prefectures, nearly all municipalities submit claims data to the SLBE, although the rates are relatively low in the Kinki region and the island prefectures, with Osaka Prefecture having the lowest percentage at 24.5%. To account for population variation across cohort and municipalities, we calculate the weighted averages for the transition probabilities by age and sex, using municipal population data from each year and the BRR from 2007 to 2018.

**Supplementary Methods 4. Monte Carlo Simulation**

In the Monte Carlo simulation (Metropolis and Ulam 1949), artificial datasets are generated by repeatedly generating random numbers that correspond to assumed probability distributions. This method allows for the calculation of statistics based on these datasets. A larger number of trials increases the precision of statistical estimates but requires more computation time.

In this study, we conducted 500,000 trials ($K=500,000)$ to generate artificial histories of LTC-status from age 65 to 94 for each sex. The process begins at age 65 for both sexes, where for each sex $s$, we generated $K$ independent random numbers $Y_{s,65,1},\ldots,Y_{s,65,K}$ from a uniform distribution of $U\left( 0,1 \right)$.

$Y_{s,65,k} \sim U\left( 0,1 \right)$ for all $k \in\left\{ 1,2,\ldots, K \right\}$

We determined the observed value for each LTC-status based on the probability distribution of each LTC-status at age 65. Let $\pi_{s,j}$ denote a $\left( 4 \times1 \right)$ vector, where the $n$th element represents the probability of being in LTC-status $x_{n}$ for sex $s$ and age $j$.

$\pi_{s,j}=\left( \pi_{s,j,1},\pi_{s,j,2},\pi_{s,j,3},\pi_{s,j,4} \right)$ where $\pi_{s,j,n}=\text{Pr}\left( x_{s,j}=x_{n} \right)$

Therefore, we categorized the outcome as no-disability, light, heavy, and death based on the probability thresholds at age 65: specifically, if the detected value is less than $\pi_{s,65,1}$, less than $\sum_{n=1}^{2} \pi_{s,65,n}$, less than $\sum_{n=1}^{3} \pi_{s,65,n}$, and$\sum_{n=1}^{3} \pi_{s,65,n}$ or greater, respectively. To determine the LTC-status of $K$ individuals at age 66, we generated$K$ independent random numbers $Y_{s,66,k} \sim U\left( 0,1 \right)$ again. For each older adult, we determined their LTC-status at age 66 based on the LTC-status at age 65 and the transition probabilities, $P_{s,66}$. Given an older adult $k$ of sex $s$ has no-disability at age 65, that is, $Y_{s,65,k}$ is less than $\pi_{s,65,1}$, we assigned their LTC-state aged 66 as no-disability, light, heavy, and death depending on whether the detected value is less than $P_{s,66}\left( x_{1},x_{1} \right)$, less than $\sum_{n=1}^{2} P_{s,66}\left( x_{1},x_{n} \right)$, less than $\sum_{n=1}^{3} P_{s,66}\left( x_{1},x_{n} \right)$, and $\sum_{n=1}^{3} P_{s,66}\left( x_{1},x_{n} \right)$ or greater, respectively. This process is repeated iteratively until age 94 for each sex, generating artificial histories of LTC-status from age 65 to 94 for each individual. After creating artificial datasets of LTC-status for $K$ individuals aged 65 to 94 for each sex, we calculated the rates of individuals in each LTC-status category and the average probability of death from the datasets.

**Supplementary Methods 5. Robustness of Estimated Transition Probabilities**

Even when examining among the seven levels of care rather than solely focusing on LTC status, a high persistence of care levels was observed. In the SLBE^4^, the MHLW reported the number of recipients who received LTCI services continuously for one year, categorized by level of care. Supplementary Table 1 displays the distribution of recipients who received LTCI services continuously for one year, based on data collected from each month between April 2012 to March 2013. As illustrated in Supplementary Table 1, recipients at any level of care are most likely to remain at the same level when receiving LTCI services for one year. When changes in care level occur, only a small proportion of recipients transitions to significantly different levels of care. For example, among recipients with light LTC status, 2.1% of SL1 recipients move to CL2, while 0.24% of CL2 recipients move to SL1. This trend has been consistently observed across all years from 2006 to 2018 and is evident for both men and women.

**Supplementary Figure 1. Flowchart of how to estimate transition probabilities of moving from one state to another across two periods**

Abbreviation: LTC-status, long-term care status.

**Supplementary Figure 2. Rates of municipal populations providing long-term claims data by prefecture in Japan.**

Note: This figure shows the rates of the total population aged 65 years and older for which municipalities provide long-term care claims data to the SLBE, conducted by the MHLW, in each prefecture.

**Supplementary Figure 3. LTC-status by age and sex (raw data)**

Abbreviation: LTC-status, long-term care status; SL1, support-required level 1; CL2, care-required level 2; CL3, care-required level 3; CL5, care-required level 5.

**Supplementary Table 1. Transition probabilities of recipients continuously receiving LTCI services for one year**

Abbreviation: SL, support-required level; CL, care-required level.

**References**

1. Chung KL. Markov Chains with Stationary Transition Probabilities. Springer-Verlag. 1967.
2. Metropolis, N., & Ulam, S. The Monte Carlo Method. *Journal of the American Statistical Association*. 1949; 44(247): 335–341. <https://doi.org/10.1080/01621459.1949.10483310>.
3. Robinson J. A Long-Term Care Status Transition Model. In: The Old-Age Crisis—Actuarial Opportunities: The 1996 Bowls Symposium. Georgia State University. 1996; 72–79.
4. Ministry of Health, Labour and Welfare (MHLW). Survey of Long-term Care Benefit Expenditures. https://www.e-stat.go.jp/stat-search/files?page=1&toukei=00450049&result_page=1(in Japanese). Downloaded on October 25, 2024.
